# Can El Niño–Southern Oscillation Increase Respiratory Infectious Diseases in China? An Empirical Study of 31 Provinces

**DOI:** 10.3390/ijerph19052971

**Published:** 2022-03-03

**Authors:** Qingyun Tang, Ke Gong, Li Xiong, Yuanxiang Dong, Wei Xu

**Affiliations:** 1 School of Economics and Management, Chongqing Jiaotong University, Chongqing 400074, China; e.mmg@163.com (Q.T.); xiongli1430602027@163.com (L.X.); 2 School of Economics and Management, Taiyuan University of Technology, Taiyuan 030006, China; dongyuanxiang@tyut.edu.cn; 3 School of Business, Jiangnan University, Wuxi 214122, China; xuwei@jiangnan.edu.cn

**Keywords:** respiratory infectious diseases, El Niño–Southern Oscillation, climate change, per capita disposable income, average years of education

## Abstract

Respiratory infectious diseases (RID) are the major form of infectious diseases in China, and are highly susceptible to climatic conditions. Current research mainly focuses on the impact of weather on RID, but there is a lack of research on the effect of El Niño–Southern Oscillation (ENSO) on RID. Therefore, this paper uses the system generalized method of moments (SYS-GMM) and the data of 31 provinces in China from 2007 to 2018 to construct a dynamic panel model to empirically test the causality between ENSO and RID morbidity. Moreover, this paper considers the moderating effects of per capita disposable income and average years of education on this causality. The results show that ENSO can positively and significantly impact RID morbidity, which is 5.842% higher during El Niño years than normal years. In addition, per capita disposable income and average years of education can effectively weaken the relationship between ENSO and RID morbidity. Thus, this paper is of great significance for improving the RID early climate warning system in China and effectively controlling the spread of RID.

## 1. Introduction

According to the World Health Organization (WHO) report [1], respiratory infectious diseases (RID) are the primary infectious diseases in the world. At present, controlling the spread of RID is still a significant public health problem in China. In 2020, 779,556 cases of notifiable respiratory infectious diseases were reported in China [2]. Respiratory infectious diseases are caused by bacterial or viral pathogens [3,4], and climate change can affect the occurrence and deterioration of RID by changing the activity and spread of viruses and bacteria and changing the immune response of vectors and hosts [5,6]. Therefore, studying the impact of climate change on the morbidity of RID can provide a basis for the prevention and treatment of RID.

In terms of global climate variability, El Niño–Southern Oscillation (ENSO) is the most crucial interannual climate variability mode [7,8]. ENSO is a coupled ocean–atmosphere natural phenomenon in the equatorial Pacific [9], which has a significant impact on the climate of China and even causes extreme weather and natural disasters [10,11,12,13]. Meteorologically, the warmer period of the ENSO is often called El Niño, and the colder period is called La Niña [14]. Furthermore, ENSO has been proven to impact the morbidity of infectious diseases, such as dengue fever, diarrhea, and malaria [15,16,17]. For example, Xiao et al. [16] found that El Niño may have contributed to the dengue fever epidemic from 1995 to 2010 in Guangdong, China. However, there is little discussion about the relationship between ENSO and the morbidity of RID in China. Therefore, this paper explores the link between ENSO and the morbidity of RID, filling this research gap.

In addition to climate change, previous studies have shown that many socio-economic factors, such as income [6,18,19,20] and education level [21,22,23], are significantly related to the RID epidemic. On the one hand, living in high-income areas means better living conditions and medical resources, which can resist the spread of RID [18,24]. For example, Wang et al. [18] pointed out that the disposable income per capita from 2013 to 2016 was negatively correlated with the morbidity of respiratory infectious diseases in mainland China. On the other hand, people in areas with higher education levels can better understand and use health-related information to prevent RID more effectively [25]. For example, Yu et al. [21] found that education level was negatively correlated with RID morbidity in 10 cities in China. However, in the face of frequent El Niño events, can high income and high education level reduce the risk of contracting respiratory infectious diseases? Unfortunately, as far as we know, existing research does not discuss these issues. Therefore, this paper studies the moderating effects of per capita disposable income and average years of education on the morbidity of ENSO and RID, which fills this research gap.

Overall, this paper conducts an empirical study on the causation between ENSO and RID morbidity, using the data of 31 provinces in China and establishing a panel model with the system generalized moment method (SYS-GMM). Secondly, this research considers the moderating effect of per capita disposable income on the relationship between ENSO and the morbidity of RID. Thirdly, this research examines the moderating effect of average years of education on the link between ENSO and RID morbidity.

This research makes the following contributions. Firstly, ENSO is the most crucial interannual climate variability mode, and past research focused on the impact of ENSO on other infectious diseases, such as dengue fever, diarrhea, and malaria [15,16,17]. Despite RID being a primary infectious disease in China, there are no studies in the literature discussing the causal relationship between ENSO and RID. Therefore, this study explores the causation between ENSO and RID morbidity in China, filling this research gap. This research provides a new perspective for climate warning of RID, namely, ENSO. In addition, although previous studies discussed the influence of income levels and education levels on RID prevalence [18,21], they ignored the impact of ENSO and did not consider the interaction between these factors and ENSO. Thus, this study explores the moderating effects of per capita disposable income and average years of education on the relationship between ENSO and RID morbidity, filling the gap in this research. Therefore, this research provides a basis for resource allocation and policy formulation to reduce RID risk in low-income and low-education areas of China during ENSO events.

## 2. Literature Review

### 2.1. The Impact of ENSO on Infectious Diseases

Climate change is the main threat to human health [26]. Mirsaeidi et al. [5] proposed that the most prominent health threat from climate change is RID risk. ENSO, as the most important interannual climate variability mode in the world [7], has been proven to be a main driving factor leading to adverse health consequences for humans [27]. Respiratory infectious diseases are caused by bacterial or viral pathogens, such as streptococcus pneumonia, respiratory syncytial virus, mycobacterium tuberculosis, etc. [3,4]. Climate change affects the occurrence and deterioration of infectious diseases by changing the activity and spread of climate-sensitive viruses and bacteria, changing the immune response of vectors and hosts [5,6]. For example, Xiao et al. [16] used wavelet coherence analysis and a generalized additive model (GAM) to find that ENSO may have contributed to the dengue fever epidemic from 1995 to 2010 in Guangdong of China. Demissie and Mengisitie [17] conducted a systematic review of the impact of ENSO on diarrheal diseases morbidity and pointed out that there is a significant association between diarrheal diseases and ENSO. Wirasatriya et al. [15] used the Pearson correlation method to prove that ENSO affects the interannual changes in the morbidity of dengue fever and malaria in Indonesia. Although RID are also climate-sensitive diseases caused by the spread of viruses and bacteria, only a few studies discuss the relationship between ENSO and RID in other countries. For example, Zaraket et al. [28] found that the peak of influenza activity in Japan was related to the warm period of ENSO using Fisher’s exact probability test and Scheffe’s multiple comparison method. Respiratory infectious diseases are the leading cause of morbidity and mortality in China, but there is no research discussing the impact of ENSO on respiratory infectious diseases in China. Therefore, it is necessary to study the relationship between ENSO and RID in China, providing a new direction for controlling the epidemic of RID.

Given this background, we put forward the following hypothesis.

**Hypothesis** **1:** *ENSO has a positive and significant impact on RID morbidity*.

### 2.2. The Moderating Effect of Income Factors

According to Grossman’s health production function model [29], income is one of the main determinants of human health. Many studies have proven the close relationship between the morbidity and spread of respiratory infectious diseases and income level [6,18,19,20]. For example, Wang et al. [18] found that the per capita disposable income from 2013 to 2016 was negatively correlated with the morbidity of respiratory infectious diseases in mainland China, establishing a geographically weighted regression model. Still, they did not consider the impact of climate change. High income usually means high social status, better living conditions, and access to more medical services, resisting RID caused by climate change [18,24]. Therefore, at the regional level, compared with people of lower per capita disposable income, people with higher per capita income are more resistant to the risk of increased morbidity of RID caused by ENSO. In other words, the higher the per capita disposable income, the more it can curb the positive relationship between ENSO and the morbidity of RID.

Based on the above background, this paper gives the following hypothesis.

**Hypothesis** **2:** *Per capita disposable income negatively moderates the relationship between ENSO and RDI morbidity (i.e., higher per capita disposable income can weaken the impact of ENSO on RID morbidity)*.

### 2.3. The Moderating Effect of Education Factors

Education factors are also related to the occurrence of respiratory infectious diseases. Gurgel et al. [22] pointed out that the low education level of a father is a risk factor for lower respiratory tract infection hospitalization in children in Brazilian using a stepwise logistic regression model. Hossain et al. [6] concluded in their periodic review report that even though a large amount of the literature has explored the link between education factors and RID, few studies exist on the moderating effect of education factors on RID and climate change. Yu et al. [21] used a multivariate meta-analysis model to analyze the morbidity of RID in 10 cities in China. They found that people with lower education levels have an increased risk of RID infection caused by extreme temperatures. Carreras et al. [23] hold that a high education level can negatively moderate the link between air temperature and respiratory infectious diseases in Argentina using a generalized additive model. On the one hand, people with higher education levels can better understand and use RID prevention and health-related information [25]. On the other hand, Lee et al. [30] pointed out that poorly educated people have less awareness of climate change risk, while highly educated people are more aware of it. Therefore, compared with residents in the area with higher average years of education, residents with lower average years of education may have insufficient knowledge of respiratory infectious diseases and ENSO and a poorer willingness to prevent and treat them. In other words, the higher the average years of education, the more the positive relationship between ENSO and morbidity of RID can be weakened.

Based on the above background, we put forward the following hypotheses.

**Hypothesis** **3:** *Average years of education negatively moderates the relationship between ENSO and RID morbidity (i.e., higher average years of education can weaken the impact of ENSO on RID morbidity)*.

## 3. Data

### 3.1. Study Area

**Figure 1:**
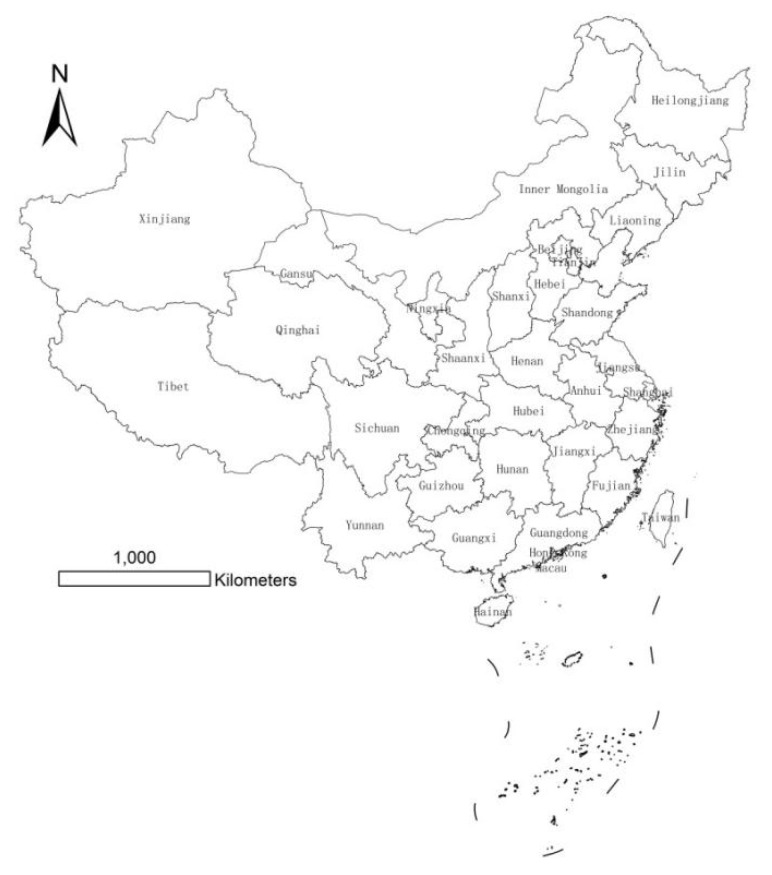
Study area (data of Hong Kong, Macau and other regions are unobtainable).

Figure 1 shows the study area of 31 provinces in mainland China in this study.

### 3.2. Variables

This paper uses panel data, which are annual data of 31 provinces in China from 2007 to 2018.

#### 3.2.1. Dependent Variables

RID morbidity ( $RID_{i,t}$ ) represents the new cases caused by RID per 100,000 population per year. We collected data on the morbidity of RID in 31 provinces in China from the China Notifiable Disease Database (https://www.phsciencedata.cn/Share (accessed on 12 March 2021)) for all available years (from 2007 to 2018). RID are Category AB respiratory infectious diseases determined in accordance with the legal diagnostic criteria and management principles of infectious diseases issued by the National Health Commission of the People’s Republic of China, including pneumonia, H1N1 influenza, tuberculosis, measles, diphtheria, scarlet fever and pertussis.

#### 3.2.2. Core Independent Variables 

The ENSO index ( $Ni\tilde{n}o1+2_{t}$ ): At present, many studies have developed a large number of ENSO indices to monitor the state changes in ENSO, mainly including *Niño*1 + 2, *Niño*3, *Niño*4, and the Southern Oscillation Index (SOI) [27]. The measurement area of the *Niño*1 + 2 index is located in the eastern Pacific region (0°–10° S, 90°–80° W) [8]. The interannual sea surface temperature variation of *Niño*1 + 2 is very obvious, even though its sea area is relatively minor compared with the sea areas of other indices [8,9,31]. In addition, some studies have shown that the *Niño*1 + 2 index is a powerful indicator of cyclical oscillations strongly correlated with the climate of China [9,31]. Therefore, we obtained the monthly *Niño*1 + 2 index data from the National Oceanic and Atmospheric Administration database (https://psl.noaa.gov/gcos_wgsp/Timeseries/Nino12/ (accessed on 27 May 2021)). We calculated the interannual monthly *Niño*1 + 2 index using a 12-point running average. 

Income level ( $In_{i,t}$ ): The income level of this article is measured by per capita disposable income, because compared with total revenue, per capita disposable income can better reflect the impact of changes in actual income levels on human health [18]. We collected the data of per capita disposable income from the China Statistical Yearbook to measure the income level of residents.

Education level ( $Edu_{i,t}$ ): The first choice for measuring the education level of a country or region is the average years of education [32]. Therefore, we collected the data of the number of people in various education levels from the China Population and Employment Statistical Yearbook. This paper uses the statistical method of the China Population and Employment Statistical Yearbook to calculate the average years of education. According to the current Chinese education system, the ratio of the educated population to the current population is calculated using the weights of 0 for illiteracy, 6 for elementary school, 9 for junior high school, 12 for high school, and 16 for college and above, representing the average years of education [33].

#### 3.2.3. Control Variables

This article controls other potential confounding factors in social and environmental aspects, which may bias the relationship between ENSO and RID morbidity. 

Urbanization rate ( $Ur_{i,t}$ ): The urbanization rate, expressed by the ratio of the urban population to the rural population, reflects the degree of urbanization in a region. Some studies believe that urbanization will affect the spread of RID [34]. To control the impact of urbanization, we collected the urbanization rate data from the China Population and Employment Statistical Yearbook.

Population age structure ( $PAS_{i,t}$ ): Some studies believe that the age structure of the social population will have an impact on the morbidity of RID [35]. The old-age dependency ratio, which refers to the ratio of the population aged 65 and above to the population aged 15–64, is usually used to measure the population age structure [36]. Therefore, we collected data on the old-age dependency ratio from the China Statistical Yearbook to control the impact of the population age structure.

Environment ( $E{nv}_{i,t}$ ): Previous studies found that ambient air pollution may increase the morbidity of respiratory infectious diseases [37]. Therefore, this paper collects data on the proportion of days in which air quality met the Grade II standard of the national ambient air quality standards (NAAQS) in one year from the China Environmental Statistical Yearbook, in order to control the environmental impact.

Medical resources ( $Med_{i,t}$ ): Local fiscal health expenditure per capita is an important indicator to measure the accessibility of medical and health resources in a region. The higher the local financial health expenditure per capita, the more abundant medical resources and the better the medical and sanitary conditions in an area, and the lower the morbidity of respiratory infectious diseases [38]. We collected the per capita local financial health expenditure from the China Statistical Yearbook to control the impact of medical resources.

### 3.3. Descriptive Statistics

Table 1 shows the summary statistics of these variables. Table 2 shows the Pearson’s correlation matrix of these variables. The results of Pearson’s correlation matrix show that the *Niño*1 + 2 index, per capita disposable income, average years of education and other control variables are all significantly correlated with RID morbidity. 

## 4. Model

### 4.1. Model Setting

Considering that the spread of respiratory infectious diseases is dependent [39], that is, RID morbidity in the previous year may affect RID morbidity in the current year, this paper introduces the one-period lag of RID morbidity (i.e., the RID morbidity of the previous year) as a control variable to examine the dynamics of RID morbidity [40]. In addition, considering that the magnitude of the morbidity of RID is much greater than that of the *Niño*1 + 2 index, to reduce the effect of magnitude and heteroscedasticity [41], we performed logarithmic conversion of the morbidity of RID. Thus, as mentioned above, considering the possible impact of ENSO on RID, the following measurement model 1 is constructed:

(1) $$ln\;RID_{i,t}=\alpha_{1}\;ln\;RID_{i,t}-1+\alpha_{2}Ni\tilde{n}o1+2_{t}+\mu_{i}+\gamma_{t}+\varepsilon_{i,t}$$

where $RID_{i,t}$ is the RID morbidity of the province *i* in time *t*, $RID_{i,t-1}$ is the one-period lag of the RID morbidity of the province *i* in time *t*, $Ni\tilde{n}o1+2_{t}$ is the *Niño*1 + 2 index in time *t*, *µ_i_* is the unobservable individual fixed effect, *γ_t_* is the unobserved time fixed effect, and *ε_i,t_* is the error term.

According to that mentioned above, income level, education level, and other social and environmental factors impact the morbidity of RID. Based on the previous variable selection above, we gradually added per capita disposable income, average years of education, and other control variables into measurement model 1. Considering that the magnitudes of per capita disposable income and average years of education are much greater than that of the *Niño*1 + 2 index, we performed logarithmic conversion of per capita disposable income and average years of education, and then constructed measurement model 2:

(2) $$\begin{array}{cc} ln\;RID_{i,t} & =\alpha_{1}\;ln\;RID_{i,t}-1+\alpha_{2}Ni\tilde{n}o1+2_{t}+\alpha_{3}\;ln\;In_{i,t}+\alpha_{4}\;ln\;E{du}_{i,t}\\ & +\alpha_{k}\sum_{k=5}^{8}{Control}_{k(i,t)}+\mu_{i}+\gamma_{t}+\varepsilon_{i,t} \end{array}$$

where $In_{i,t}$ is the per capita disposable income of the province *i* in time *t*, $Edu_{i,t}$ is the average years of education of the province *i* in time *t*, and ${Control}_{k(i,t)}$ represents the control variables of urbanization rate ( $Ur_{i,t}$ ), population age structure ( $PAS_{i,t}$ ), environment ( $E{nv}_{i,t}$ ), and medical resources ( $Med_{i,t}$ ).

Considering the moderating effect of income level and education level on the relationship between ENSO and the morbidity of RID, we introduced the interaction item of *Niño*1 + 2 index and per capita disposable income and the interaction item of *Niño*1 + 2 index and per capita education years, and constructed the following measurement model 3 and 4:

(3) $$\begin{array}{cc} ln\;RID_{i,t} & =\alpha_{1}\;ln\;RID_{i,t}-1+\alpha_{2}Ni\tilde{n}o1+2_{t}+\alpha_{3}\;ln\;In_{i,t}+\alpha_{4}\;ln\;E{du}_{i,t}\\ & +\alpha_{k}\sum_{k=5}^{8}{Control}_{k(i,t)}+\alpha_{9}Ni\tilde{n}o1+2_{t}\times ln\;In_{i,t}+\mu_{i}+\gamma_{t}+\varepsilon_{i,t} \end{array}$$

(4) $$\begin{array}{cc} ln\;RID_{i,t} & =\alpha_{1}\;ln\;RID_{i,t}-1+\alpha_{2}Ni\tilde{n}o1+2_{t}+\alpha_{3}\;ln\;In_{i,t}+\alpha_{4}\;ln\;E{du}_{i,t}\\ & +\alpha_{k}\sum_{k=5}^{8}{Control}_{k(i,t)}+\alpha_{10}Ni\tilde{n}o1+2_{t}\times \;ln\;Edu_{i,t}+\mu_{i}+\gamma_{t}+\varepsilon_{i,t} \end{array}$$

where $Ni\tilde{n}o1+2_{t}\times ln\;In_{i,t}$ is the interaction item of the *Niño*1 + 2 index and per capita disposable income of the province *I* in time *t*, and $Ni\tilde{n}o1+2_{t}\times ln\;Edu_{i,t}+\mu i+\gamma t+\varepsilon i,t$ is the interaction item of the *Niño*1 + 2 index and per capita education years of the province *i* in time *t*.

### 4.2. Endogenous Analysis

First, some unobservable variables may be missed, which are included in the error term in the model. If the independent variables are correlated with these missing variables in the model, it will lead to endogenous problems, leading to errors in the estimation results. Using the generalized method of moments (GMM) to construct a panel model can eliminate errors caused by missing variables [42]. 

Secondly, another source of bias is the assumption that RID morbidity is static. However, due to the persistence of RID, the current morbidity depends on the previous morbidity [39], so it is more reasonable to establish a dynamic panel model. There are two GMM estimators for constructing a dynamic panel model: the difference GMM (DIF-GMM) and the system generalized method of moments (SYS-GMM) [43,44]. Blundell and Bond [44] proved that the SYS-GMM estimator has fewer biases and higher accuracy than the DIF-GMM estimator. Thus, we chose the SYS-GMM estimator and added RID morbidity with a lag of one period in all models, while using two later period-lagged RID morbidity indexes as instruments in the model. At the same time, we also used no more than two period-lagged *Niño*1 + 2 indexes as instruments in all models. In addition, we added time fixed effects into all models to control the unobservable time effect, which can eliminate the deviation caused by time changes.

Thirdly, the Hansen test and Arellano–Bond (AR) test can check the consistency of the SYS-GMM estimation results. Accepting the null hypothesis (the instruments are exogenous) of the Hansen test indicates that the instruments of the SYS-GMM model are valid. Accepting the null hypothesis (there is no second-order autocorrelation in the residual) of the AR (2) test represents that the estimation result of SYS-GMM is reliable [40,43,44]. Moreover, SYS-GMM includes two forms, which are one-step and two-step methods. Theoretically, because the two-step method uses the best weight matrix, the estimation results of the two-step method are more accurate than those of the one-step method [40]. Therefore, this research adopts the two-step form of SYS-GMM.

Finally, due to the potential endogeneity issues mentioned above, compared with SYS-GMM, standard ordinary least squares (OLS) and fixed effects (FE) panel models are not applicable. Nevertheless, the robustness of the SYS-GMM estimated results could be judged through OLS and FE [45]. The detailed robustness analysis is further elaborated in Section 5.4.

### 4.3. Granger Causality Test

Before estimating the model using SYS-GMM, this paper utilizes the panel Granger causality test, using *Niño*1 + 2 index and RID morbidity, to examine whether ENSO causes the occurrence of RID. The Granger causality test method was proposed by Granger [46], and is mostly applied to time series data. When the data type is extended to panel data, due to the homogeneous or heterogeneous relationship between different cross-sections, the causal relationship between panel data depends on whether there is a one-to-one causal relationship between the entire cross-sections. Therefore, this paper adopts the panel Granger causality test method based on the cross-sectional Wald statistic proposed by Dumitrescu and Hurlin [47], which can more accurately explain whether there is a causal relationship between panel data. The results of the panel Granger causality test between ENSO and RID are shown in Table 3. The results show that ENSO Granger causes RID (*p* < 0.01), but RID does not show that Granger causes ENSO (*p* > 0.1).

## 5. Model Results and Discussion

### 5.1. Does ENSO Have a Significant and Positive Impact on RID Morbidity?

To explore Hypothesis 1, suggesting that ENSO can impact RID morbidity, we first performed SYS-GMM estimation on model 1. Secondly, by the stepwise addition of per capita disposable income, average years of education, and other social and environmental control variables into model 1, we constructed model 2. Columns (1)–(7) of Table 4 show the SYS-GMM results of the stepwise addition of the control variables. The SYS-GMM model accepts the null hypothesis of the Hansen and AR (2) tests, meaning that the instruments are valid and there is no second-order autocorrelation problem [40]. 

The estimated results from columns (1) to (7) in Table 4 indicate that the *Niño*1 + 2 index is significantly and positively correlated with RID morbidity. In column (7), for every increase of 1 in the *Niño*1 + 2 index, the RID morbidity increases by 3.769% through calculation (the exact calculation principle is displayed in formula (A4) in Appendix A). Although there is currently no study on ENSO and respiratory infectious diseases in China, Xiao et al. [16] pointed out that ENSO contributed to the dengue fever epidemic. In addition, Zaraket et al. [28] proposed that the peak of influenza activity in Japan was related to the warm period of ENSO. Our research results are consistent with theirs. 

Moreover, RID morbidity caused by ENSO is 9.691% higher during El Niño years compared with La Niña years, and RID morbidity caused by ENSO is 5.842% higher during El Niño years compared with normal years (the detailed calculation method is displayed in formula (A5) and formula (A6) in Appendix A). The strong El Niño event from 2015 to 2016 caused global disease outbreaks. The intensity of infectious disease activities in the U.S., Brazil, Southeast Asia, Tanzania, and other regions was 2.5–28% higher during El Niño years compared with non-El Niño years [48]. Our research results are relatively consistent with theirs. Furthermore, the intense El Niño event in 2015–2016 caused catastrophic weather in China, such as severe drought and extreme temperatures [13], which affect the spread of viruses and bacteria and the immune response of the vector and host [5]. Thus, our results provide direct evidence that El Niño created the ecological conditions for the expansion of RID in China.

In addition, columns (2) to (7) of Table 4 show that per capita disposable income and average years of education are both significant and negative, which indicates that the increase in income level and education level can reduce RID morbidity. These results are consistent with [18,21]. In addition, the relationship between other control variables and RID morbidity was not statistically significant, indicating that these confounding factors have no significant impact on RID morbidity, so we did not discuss them in this article.

### 5.2. Does Per Capita Disposable Income Have a Moderating Effect on the Relationship between ENSO and RID Morbidity?

To explore Hypothesis 2 that per capita disposable income can negatively moderate the impact of ENSO on RID morbidity, this paper accounts for the interaction term between the *Niño*1 + 2 index and per capita disposable income, which is the model 3 mentioned above. Column (1) of Table 5 shows the SYS-GMM results of adding the interaction term between the *Niño*1 + 2 index and per capita disposable income. The coefficient of the interaction term between the *Niño*1 + 2 index and per capita disposable income is significant and negative, opposite to the coefficient of the single *Niño*1 + 2 index. The results indicate that the positive impact of ENSO on RID morbidity diminishes with the increase in per capita disposable income.

**Figure 2:**
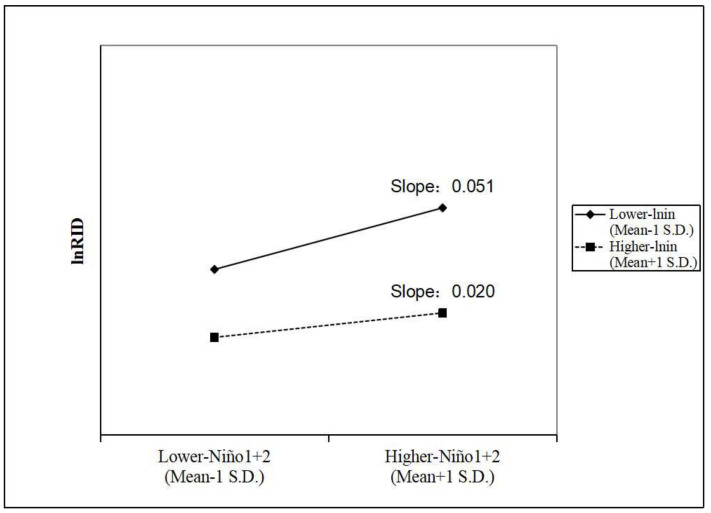
The moderating effect of per capita disposable income on the relationship between ENSO and RID morbidity.

In addition, Figure 2 visually shows the moderating effect of per capita disposable income. For every increase of 1 in the *Niño*1 + 2 index, the RID increases by 5.232% when per capita disposable income is lower, but the RID only increases by 2.02% when the per capita disposable income is higher (the exact calculation principle is displayed in formula (A4)).

Past studies have shown that high-income levels can reduce RID morbidity in China [18]. However, they did not consider the combined effects of income levels and climate change on RID morbidity. Our results provide additional evidence of the moderating effect of per capita disposable income. On the one hand, people with higher income generally have better living conditions [18], helping them be less exposed to the unhealthy environments caused by ENSO. On the other hand, they have more opportunities for quality medical care and health insurance [24], avoiding the risk of RID transmission during ENSO events.

### 5.3. Does Average Years of Education Have a Moderating Effect on the Relationship between ENSO and RID Morbidity?

To verify Hypothesis 3, suggesting that average years of education can negatively moderate the impact of ENSO on RID morbidity, this paper accounts for the interaction term between the *Niño*1 + 2 index and average years of education, which is the measurement model 4 mentioned above. Column (2) of Table 5 shows the SYS-GMM results of adding the interaction term between the *Niño*1 + 2 index and average years of education. The result indicates that the interaction term between the *Niño*1 + 2 index and average years of education is significantly and negatively correlated with RID morbidity, which is opposite to the coefficient of the single *Niño*1 + 2 index. The results prove that the increase in average years of education can weaken the positive impact of ENSO on RID morbidity. 

**Figure 3:**
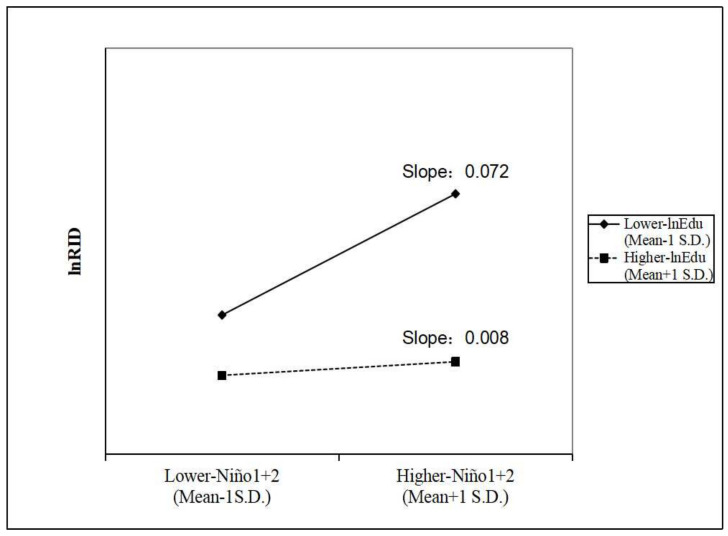
The moderating effect of average years of education on the relationship between ENSO and RID morbidity.

Moreover, Figure 3 intuitively displays the moderating effect of average years of education. For every increase of 1 in the *Niño*1 + 2 index, the RID increases by 7.466% when average years of education are lower. However, the RID only increases by 0.803% when average years of education are higher (the exact calculation principle is displayed in the formula (A4)).

Yu et al. [21] pointed out that lower education levels have an increased risk of RID infection caused by extreme temperatures in China. In that way, can higher education levels also resist the RID risk caused by ENSO? Our answer is yes. Since ENSO is the most critical climate variability mode, which leads to extreme temperatures and other severe weather events, such as drought and extreme precipitation [10,11,12,13], our research results more comprehensively characterize the moderating effect of education level on the relationship between climate change and RID. Facing ENSO, people with high education levels have a relatively strong awareness of disease prevention and a better understanding and use of health-related information, enabling them to protect themselves during RID epidemics [25,50]. More importantly, they also have higher climate literacy and more intensive climate change risk awareness [30], which improves their ability to deal with RID prevalence when ENSO strikes. 

### 5.4. Robustness Analysis

Bond [45] put forward that when the estimated coefficient of the lagged dependent variable of SYS-GMM is between the estimated coefficients of OLS and F.E., the SYS-GMM estimator is robust. Thus, we also adopt OLS and FE to re-estimate models 3 and 4. Columns (1) to (3) of Table 6, respectively, show the estimation results using OLS, FE and SYS-GMM for model 3. Columns (1) to (3) of Table 7, respectively, show the estimation results using OLS, FE and SYS-GMM for model 4. The results in column (3) of Table 6 and column (3) of Table 7 show that the SYS-GMM estimated coefficient of the one-period lagged morbidity of RID is, respectively, 0.756 and 0.764, which is between the estimated values of OLS and FE. These results show that the above estimation results are robust.

## 6. Conclusions

By using the data of 31 provinces in China from 2007 to 2018 and applying a dynamic panel data model and SYS-GMM, this paper explores the causation of ENSO on RID morbidity. In addition, this paper considers the moderating effects of per capita disposable income and education on the relationship between ENSO and RID morbidity. The conclusions of this empirical analysis are as follows:

First, ENSO has a positive impact on RID morbidity in China. For every increase of 1 in the *Niño*1 + 2 index, the RID morbidity increases by 3.769%. In addition, compared with La Niña years and normal years, RID morbidity caused by ENSO is, respectively, 9.691% and 5.842% higher during El Niño years. RID is one of the most critical public health problems in China. However, current research has not discussed the impact of ENSO on RID in China. Therefore, we provide a new perspective for RID climate warning in China, namely, ENSO. This article advocates that the Chinese meteorological system and public health system should strengthen the early warning of ENSO. Based on the predictability of ENSO [51], the advanced deployment of prevention and control measures and the storage of medical supplies before ENSO events will play a key role in controlling the spread and expansion of RID.

Second, higher per capita disposable income can mitigate the positive impact of ENSO on RID morbidity. RID increases by 5.232% for every increase of 1 in the *Niño*1 + 2 index in lower-income areas, while RID only increases by 2.02% in higher-income areas. The rise in income allows people to escape from the harsh ecological environment before the ENSO event and to prepare better living conditions and resources for preventing RID [18,24]. Therefore, we suggest that areas with relatively low income levels should pay more attention to changes in ENSO and be allocated more medical resources while improving their economic statuses.

Third, higher average years of education can weaken the relationship between ENSO and RID morbidity. For every increase of 1 in the *Niño*1 + 2 index, RID morbidity increases by 7.466% in areas with lower education levels, but only by 0.803% in areas with higher education levels. The improvement in education levels for the public can promote the mastery and application of RID prevention knowledge and improve the ability to perceive climate risks [30,50]. Thus, based on the continuous implementation of nine-year compulsory education, policymakers should also popularize RID-related knowledge and preventive measures and raise awareness of ENSO risks for less educated people, making them less vulnerable to RID when facing ENSO.

Finally, this study still has some limitations. First, due to the reported cases being symptomatic and hospitalized, the under-reporting of cases is inevitable. In the future, it will be more pertinent to conduct research under the premise of obtaining real data from hospitals. Second, the impact of air pollution on RID is critical. Since more detailed indicators of pollutants are not available at the provincial level, such as concentrations of different types of pollutants (particulate matter and toxic gas emissions) and pollutants from different sources (from factories, vehicles, or living environment), this paper uses the proportion of days in a year that meet NAAQS to measure the macro impact of air pollution on RID morbidity. In the future, it is necessary to obtain more comprehensive pollution data in a smaller area, so as to better examine the joint impact of ENSO and air pollution on the transmission of RID. Finally, the microscopic characteristics of an individual, such as smoking, high stress, low-quality food, etc., can be very harmful to an individual’s respiratory tract. Therefore, future research should be considered from a microscopic perspective.

## Figures and Tables

**Table 1:** Summary statistics of variables.

Variable	Observations	Mean	Std. Dev.	Min	Max
$RID_{i,t}$	372	162.293	76.675	54.276	626.743
$Ni\tilde{n}o1+2_{t}$	372	−0.055	0.621	−1.067	1.433
$In_{i,t}$	372	20366.5	8579.501	9740	64183
$Edu_{i,t}$	372	8.825	1.166	4.222	12.555
$Ur_{i,t}$	372	1.586	1.612	0.273	8.623
$PAS_{i,t}$	372	13.105	2.946	6.7	22.7
$E{nv}_{i,t}$	372	0.781	0.17	0.134	1
$Med_{i,t}$	372	705.097	443.279	93.155	3020.622

**Table 2:** Pearson’s correlation matrix of variables.

	$RID_{i,t}$	$Ni\tilde{n}o1+2_{t}$	$ln\;In_{i,t}$	$ln\;Edu_{i,t}$	$Ur_{i,t}$	$PAS_{i,t}$	$E{\mathrm{nv}}_{i,t}$	$ln\;Med_{i,t}$
$RID_{i,t}$	1.000							
	(-)							
$Ni\tilde{n}o1+2_{t}$	0.092 *	1.000						
	(0.076)	(-)						
$ln\;In_{i,t}$	−0.622 ***	0.208 ***	1.000					
	(0.000)	(0.000)	(-)					
$ln\;Edu_{i,t}$	−0.536 ***	0.087*	0.644 ***	1.000				
	(0.000)	(0.094)	(0.000)	(-)				
$Ur_{i,t}$	−0.524***	0.038	0.719 ***	0.629 ***	1.000			
	(0.000)	(0.464)	(0.000)	(0.000)	(-)			
$PAS_{i,t}$	−0.367 ***	0.101 *	0.334 ***	0.316 ***	0.162 ***	1.000		
	(0.000)	(0.051)	(0.000)	(0.000)	(0.002)	(-)		
$E{nv}_{i,t}$	−0.327 ***	−0.017	0.168 ***	0.177 ***	0.188 ***	0.107 **	1.000	
	(0.000)	(0.741)	(0.001)	(0.001)	(0.000)	(0.039)	(-)	
$ln\;Med_{i,t}$	−0.256 ***	0.386 ***	0.547 ***	0.199 ***	0.314 ***	0.084	−0.012	1.000
	(0.000)	(0.000)	(0.000)	(0.000)	(0.000)	(0.106)	(0.813)	(-)

Note: The significance test for the Pearson’s correlation coefficient is the *t*-test, for which the null hypothesis is no relationship between the two variables, and corresponding *p*-values are in parentheses. Significance levels are *** *p* < 0.01, ** *p* < 0.05, and * *p* < 0.1.

**Table 3:** The panel Granger causality test results between ENSO and RID morbidity.

Variable	W-Bar	Z-Bar	*p*-Value
*ENSO*→*RID*	3.619	4.507	0.000
*RID*→*ENSO*	2.3307	0.9207	0.357

Note: W-bar is the mean Wald test statistic of the panel, and Z-bar is the standardized statistic of Wald test statistic. If the W-bar is greater than the critical value of the rejection domain corresponding to the significance level *p*, the null hypothesis is rejected: ENSO is not the Granger-cause for RID (RID is not the Granger-cause for ENSO).

**Table 4:** Estimation results of the relationship between ENSO and RID morbidity by stepwise addition of control variables.

	SYS-GMM						
	(1)	(2)	(3)	(4)	(5)	(6)	(7)
$ln\;RID_{i,t-1}$	0.971 ***	0.869 ***	0.746 ***	0.788 ***	0.742 ***	0.743 ***	0.772 ***
	(0.049)	(0.084)	(0.142)	(0.163)	(0.163)	(0.165)	(0.182)
$Ni\tilde{n}o1+2_{t}$	0.028 **	0.031 ***	0.028***	0.029 ***	0.036 ***	0.035 **	0.037 ***
	(0.010)	(0.010)	(0.009)	(0.010)	(0.013)	(0.013)	(0.013)
$ln\;In_{i,t}$		−0.130*	−0.134 *	−0.130 *	−0.148 *	−0.144 *	−0.134 *
		(0.069)	(0.073)	(0.076)	(0.080)	(0.078)	(0.077)
$ln\;Edu_{i,t}$			−0.311 *	−0.316 **	−0.311 **	−0.306 **	−0.316 **
			(0.169)	(0.141)	(0.144)	(0.141)	(0.139)
$Ur_{i,t}$				0.007	0.005	0.005	0.010
				(0.012)	(0.012)	(0.012)	(0.014)
$PAS_{i,t}$					0.007	0.007	0.007
					(0.006)	(0.006)	(0.007)
$E{nv}_{i,t}$						−0.055	−0.047
						(0.064)	(0.062)
$ln\;Med_{i,t}$							−0.017
							(0.018)
Constant	0.098	1.899 *	3.241 *	2.981 *	3.477 **	3.460 *	3.326 *
	(0.259)	(1.083)	(1.601)	(1.656)	(1.685)	(1.703)	(1.830)
Observations	341	341	341	341	341	341	341
Number of states	31	31	31	31	31	31	31
Time fixed effect?	YES	YES	YES	YES	YES	YES	YES
Hansen test	0.120	0.117	0.078	0.117	0.111	0.107	0.103
Arellano-Bond test for AR (1)	0.007	0.007	0.007	0.008	0.008	0.008	0.008
Arellano-Bond test for AR (2)	0.407	0.453	0.450	0.453	0.454	0.468	0.421

Note: Columns (1)–(7) are the SYS-GMM results of the stepwise addition of the control variables. Robust standard errors in parentheses are clustered at the province level [49]. Significance levels are *** *p* < 0.01, ** *p* < 0.05, and * *p* < 0.1.

**Table 5:** Estimation results of the moderating effect of per capita disposable income and average years of education on the relationship between ENSO and RID morbidity.

	SYS-GMM	
	(1)	(2)
$ln\;RID_{i,t-1}$	0.756 ***	0.764 ***
	(0.168)	(0.161)
$Ni\tilde{n}o1+2_{t}$	0.464 *	0.519 ***
	(0.151)	(0.187)
$ln\;In_{i,t}$	−0.128 *	−0.130 *
	(0.075)	(0.074)
$ln\;Edu_{i,t}$	−0.304 *	−0.303 *
	(0.149)	(0.168)
$Ur_{i,t}$	0.006	0.007
	(0.014)	(0.012)
$PAS_{i,t}$	0.007	0.007
	(0.007)	(0.007)
$E{nv}_{i,t}$	−0.052	−0.050
	(0.062)	(0.061)
$ln\;Med_{i,t}$	−0.015	−0.012
	(0.019)	(0.019)
$Ni\tilde{n}o1+2_{t}\times ln\;In_{i,t}$	−0.044*	
	(0.036)	
$Ni\tilde{n}o1+2_{t}\times ln\;Edu_{i,t}$		−0.221 **
		(0.087)
Constant	3.324 *	3.265 *
	(1.661)	(1.646)
Observations	341	341
Number of states	31	31
Time fixed effect?	YES	YES
Hansen test	0.116	0.127
Arellano–Bond test for AR(1)	0.007	0.007
Arellano–Bond test for AR(2)	0.413	0.384

Note: Column (1) shows the SYS-GMM results of adding the interaction term between the *Niño*1 + 2 index and per capita disposable income. Column (2) shows the SYS-GMM results of adding the interaction term between the *Niño*1 + 2 index and average years of education. Robust standard errors in parentheses are clustered at the province level [49]. Significance levels are *** *p* < 0.01, ** *p* < 0.05, * *p* < 0.1.

**Table 6:** Robustness analysis considering the moderating effect of per capita disposable income (comparison of estimation results of OLS, FE, and SYS-GMM).

	(1)	(2)	(3)
	OLS	FE	SYS-GMM
$ln\;RID_{i,t-1}$	0.958 ***	0.560 ***	0.756 ***
	(0.043)	(0.098)	(0.168)
$Ni\tilde{n}o1+2_{t}$	0.395	0.405 **	0.464 *
	(0.251)	(0.010)	(0.151)
$ln\;In_{i,t}$	−0.051 *	−0.077	−0.128 *
	(0.028)	(0.051)	(0.075)
$ln\;Edu_{i,t}$	−0.185 ***	−0.651 ***	−0.304 *
	(0.061)	(0.201)	(0.149)
$Ur_{i,t}$	0.014 ***	0.084 *	0.006
	(0.003)	(0.043)	(0.014)
$PAS_{i,t}$	0.004 *	0.010 **	0.007
	(0.002)	(0.004)	(0.007)
$E{nv}_{i,t}$	−0.015	0.008	−0.052
	(0.034)	(0.042)	(0.062)
$ln\;Med_{i,t}$	−0.017	−0.047 *	−0.015
	(0.013)	(0.026)	(0.019)
$Ni\tilde{n}o1+2_{t}\times ln\;In_{i,t}$	−0.037	−0.037 *	−0.044 *
	(0.026)	(0.020)	(0.036)
Constant	1.197 **	4.874 ***	3.324 *
	(0.475)	(0.761)	(1.661)
Observations	341	341	341
Number of states	31	31	31
Time fixed effect?	YES	YES	YES
r^2^	0.931	0.692	
Hansen test			0.116
Arellano–Bond test for AR(1)			0.007
Arellano–Bond test for AR(2)			0.413

Note: Columns (1) to (3) are the estimation results using, respectively, OLS, FE and SYS-GMM for model 3. Robust standard errors in parentheses are clustered at the province level [49]. Significance levels are *** *p* < 0.01, ** *p* < 0.05, and * *p* < 0.1.

**Table 7:** Robustness analysis considering the moderating effect of average years of education (comparison of estimation results of OLS, FE, and SYS-GMM).

	(1)	(2)	(3)
	OLS	FE	SYS-GMM
$ln\;RID_{i,t-1}$	0.957 ***	0.563 ***	0.764 ***
	(0.042)	(0.096)	(0.161)
$Ni\tilde{n}o1+2_{t}$	0.434 ***	0.403 ***	0.519 ***
	(0.131)	(0.008)	(0.187)
$ln\;In_{i,t}$	−0.048 *	−0.068	−0.130 *
	(0.028)	(0.051)	(0.074)
$ln\;Edu_{i,t}$	−0.179 ***	−0.652 ***	−0.303 *
	(0.061)	(0.194)	(0.168)
$Ur_{i,t}$	0.013 ***	0.083 *	0.007
	(0.003)	(0.043)	(0.012)
$PAS_{i,t}$	0.004 *	0.010 **	0.007
	(0.002)	(0.004)	(0.007)
$E{nv}_{i,t}$	−0.016	0.006	−0.050
	(0.033)	(0.042)	(0.061)
$ln\;Med_{i,t}$	−0.015	−0.046*	−0.012
	(0.013)	(0.026)	(0.019)
$Ni\tilde{n}o1+2_{t}\times ln\;Edu_{i,t}$	−0.184 ***	−0.167 ***	−0.221 **
	(0.062)	(0.050)	(0.087)
Constant	1.154 **	4.775 ***	3.265 *
	(0.466)	(0.780)	(1.646)
Observations	341	341	341
Number of states	31	31	31
Time fixed effect?	YES	YES	YES
r2	0.932	0.696	
Hansen test			0.127
Arellano–Bond test for AR(1)			0.007
Arellano–Bond test for AR(2)			0.384

Note: Columns (1) to (3) are the estimation results using, respectively, OLS, FE and SYS-GMM for model 4. Robust standard errors in parentheses are clustered at the province level [49]. Significance levels are *** *p* < 0.01, ** *p* < 0.05, and * *p* < 0.1.

## Data Availability

The data presented in this study are available on request from the corresponding author.

## Appendix A

Due to this paper performing a natural logarithmic transformation of RID morbidity, the estimated coefficients must be converted. The conversion method takes the following formula as an example:

(A1)
$$ln\;y_{1}=a_{0}+a_{1}x$$

If $x\to x+1;y_{1}\to y_{2}$ :

(A2)
$$ln\;y_{2}=a_{0}+a_{1}(x+1)$$

Then

(A3)
$$\frac{y2}{y1}=e^{\left[a_{0}+a_{1}(x+1)-(a_{0}+a_{1}x)\right]}=e^{a_{1}}$$

(A4)
$$y_{2}-y_{1}=e^{a_{1}}y_{1}-y_{1}=(e^{a_{1}}-1)y_{1}$$

This means that a year can be classified as an El Niño year when the interannual *Niño*1 + 2 index is greater than 1, and as a La Niña year when the interannual *Niño*1 + 2 index is smaller than −0.8 [52]. For example, considering 2007 to 2018, the *Niño*1 + 2 index was 1.4325 in 2015, which is an El Niño year, and the *Niño*1 + 2 index was −1.0675 in 2007, which is a La Niña year. Moreover, the average value of the *Niño*1 + 2 index was −0.102 in normal years. Substituting them into the regression equation of column (7) in Table 4 obtains the following results:

(A5)
$$\frac{RID_{El\;Nino}-RID_{La\;Nina}}{RID_{La\;Nina}}=e^{[1.4325-(-1.0675)]\times 0.037}-1=e^{2.5\times 0.037}-1=9.691\%$$

(A6)
$$\frac{RID_{El\;Nino}-RID_{\mathrm{normal}}}{RID_{\mathrm{normal}}}=e^{[1.4325-(-0.102)]\times 0.037}-1=e^{1.5345\times 0.037}-1=5.842\%$$
