# Correction: Tang et al. Can El Niño–Southern Oscillation Increase Respiratory Infectious Diseases in China? An Empirical Study of 31 Provinces. *Int. J. Environ. Res. Public Health* 2022, *19*, 2971

**DOI:** 10.3390/ijerph19074194

**Published:** 2022-04-01

**Authors:** Qingyun Tang, Ke Gong, Li Xiong, Yuanxiang Dong, Wei Xu

**Affiliations:** 1 School of Economics and Management, Chongqing Jiaotong University, Chongqing 400074, China; e.mmg@163.com (Q.T.); xiongli1430602027@163.com (L.X.); 2 School of Economics and Management, Taiyuan University of Technology, Taiyuan 030006, China; dongyuanxiang@tyut.edu.cn; 3 School of Business, Jiangnan University, Wuxi 214122, China; xuwei@jiangnan.edu.cn

## 1. Error in Figure

In the original publication [1], there was a mistake in ***Figure 1*** as published. ***In the original publication, Figure 1 only shows the study area of this paper and does not show the complete China Map. Therefore, for the academic rigor of the paper, we modify Figure 1 to a complete map.*** The corrected ***Figure 1*** appears below.

The authors apologize for any inconvenience caused and state that the scientific conclusions are unaffected. The original publication has also been updated.

## Figures and Tables

**Figure 1:**
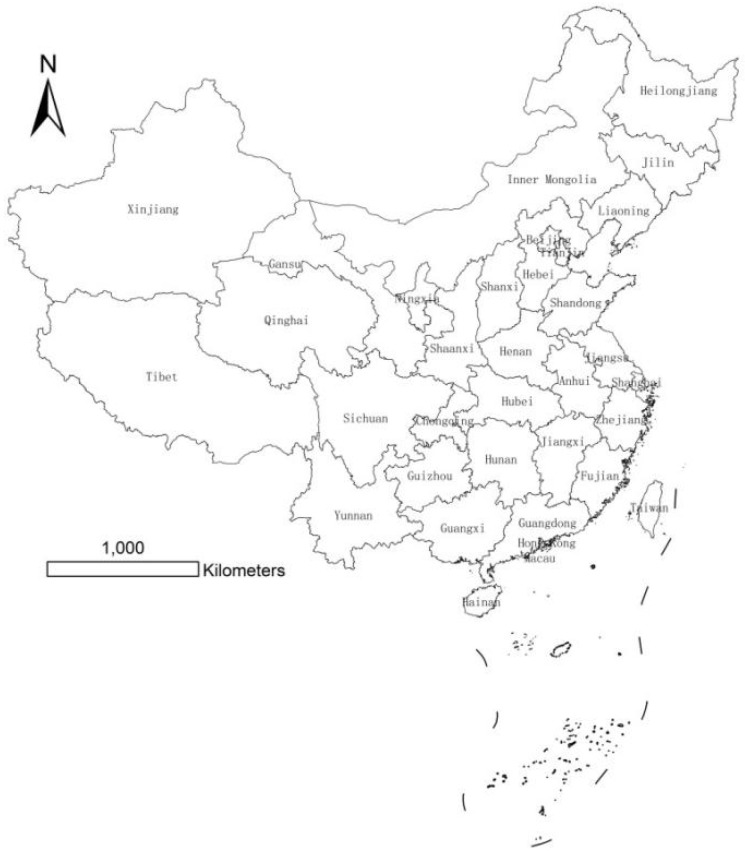
Study area (data of Hong Kong, Macau and other regions are unobtainable).
